# Willingness to participate in prevention programs for cardiometabolic diseases

**DOI:** 10.1186/s12889-015-1379-0

**Published:** 2015-01-31

**Authors:** Jessica Petter, Margreet M Reitsma-van Rooijen, Joke C Korevaar, Markus MJ Nielen

**Affiliations:** NIVEL (Netherlands Institute for Health Services Research), P.O. Box 1568, 3500 BN Utrecht, The Netherlands

**Keywords:** Cardiometabolic diseases, Health check, Lifestyle intervention, Willingness to participate

## Abstract

**Background:**

Cardiometabolic diseases are the leading cause of death worldwide and result in decreased quality of life for patients and increased healthcare costs. Population-based prevention programs may prevent the onset and development of cardiometabolic diseases. The effectiveness of these programs depends on participation rates. This study identified factors related to willingness to participate in health checks and lifestyle intervention programs to prevent cardiometabolic diseases.

**Methods:**

A questionnaire was sent to 1,500 Dutch adults, participating in the Dutch Health Care Consumer Panel of NIVEL. The questionnaire was developed by NIVEL. Predictors of willingness to participate were identified with logistic regression analyses. Predictors investigated were socio-demographic variables, risk factors for cardiometabolic diseases and motivational aspects.

**Results:**

The response rate was 63%. 56% of the participants in our study were willing to participate in a health check. Higher age was associated with increased willingness to participate, as was the desire to know the actual risk for cardiometabolic diseases (OR = 4.6). Becoming unnecessarily worried was identified as a barrier (OR = 0.3). 47% were willing to participate in a lifestyle intervention program. People aged 39–65 were most willing to participate. Attention for prevention relapse behavior (OR = 3.3), informing the general practitioner about results (OR = 2.6) and conducting the program in a group (OR = 2.0) were positively associated with willingness to participate in lifestyle interventions.

**Conclusions:**

Willingness to participate in a health check depended on personal beliefs, whereas social aspects contributed most to willingness to participate in a lifestyle intervention program. This information can be used to optimize and tailor the promotion of prevention programs.

## Background

Cardiometabolic diseases, including type II diabetes mellitus, cardiovascular diseases (CVD) and chronic kidney diseases, are the leading cause of death worldwide, accounting for about 30% of all deaths. This number is expected to increase in the next 15 years [1,2]. Besides aging of the population, an unhealthy lifestyle significantly contributes to the risk of developing cardiometabolic diseases [3-5]. These diseases result in decreased quality of life and severe complications for individuals and increased healthcare costs for society [6-8], which makes it important to prevent or delay the onset and development of cardiometabolic diseases. Population-based prevention programs might contribute to this [9,10].

Population-based prevention programs often follow a stepwise approach, including screening followed by lifestyle intervention program and/or treatment for high risk individuals [11,12]. This approach is also used in the cardiometabolic prevention consultation program in Dutch primary care [13,14]. The program starts with a health check, consisting of a short (online) questionnaire about current lifestyle, age and gender, to estimate the risk on cardiometabolic diseases. For individuals at high risk, the next step is a lifestyle intervention program, for example aimed at smoking cessation and physical exercise. The effectiveness of such a prevention program at population level is largely determined by the participation rate, which is in general low [15,16]. According to a recently published review of Koopmans et al., there is a large variation in participation rates in health checks and lifestyle intervention programs. Median response rates of 33% were found for health checks as well as for lifestyle intervention programs [17,18]. These figures demonstrate that there is ample room for improvement in participation rates. To increase these rates, it is important to know which factors influence willingness to participate.

Several studies identified factors related to participation. Participants in health checks are in general older, female, have a higher education level and are more often non-smoker compared to non-participants [17,19,20]. Practical aspects that restrained from participation in health checks are already receiving medical treatment and time investment, as are personal beliefs such as fear of the outcome of the check and feeling healthy [14,20,21]. Participants in lifestyle intervention programs are in general older, higher educated and have a worsened risk profile [15,22-24]. Practical restraining factors for participation in a lifestyle program are already receiving medical treatment, time investment, travel distance and costs [15,22,25]. Personal beliefs, such as willingness to change lifestyle and awareness of an unhealthy lifestyle or poor health, were motivating to participate. Low risk perception and low self-efficacy were restraining for participation [15,23,24,26]. Murray et al. identified formal support of care providers and conducting exercise activities in a group as most relevant social factors related to participation, which are confirmed as motivating factors in other studies [8,27,28].

These results are from studies performed among high risk individuals or patients suffering from cardiometabolic diseases, especially in studies about lifestyle intervention programs. However, the target population in the prevention of cardiometabolic diseases are people without these diseases. The willing to participate in prevention programs of these people is a precondition for effective prevention. It is still unknown which (type of) factors are most related to willingness to participate. Therefore, the aim of this study is to identify factors that influence willingness to participate in health checks and lifestyle intervention programs for cardiometabolic diseases in adults without cardiometabolic diseases.

## Methods

### Study population and measurements

Data was collected in the Dutch Health Care Consumer Panel of NIVEL (Netherlands Institute for Health Services Research). The panel contains a dynamic population of 6,000 members aged 18 years and older [29]. The members are representative for the general population in the Netherlands regarding age and gender. A questionnaire was sent to a random sample of 1,500 members. The questionnaire was developed by NIVEL and was based on existing questionnaires about participation in prevention programs. Participants already suffering from cardiometabolic diseases were excluded from analyses. Protection of the collected data was registered with the Dutch Data Protection Authority (nr. 1262949) [29]. According to Dutch legislation, neither obtaining informed consent nor approval by a medical ethics committee was obligatory for this study (http://www.ccmo.nl/en/your-research-does-it-fall-under-the-wmo).

### Outcome variables and predictors

The two outcome variables were willingness to participate in a health check and willingness to participate in a lifestyle intervention program. Independent variables were socio-demographic variables (age, gender, education level and marital status), risk factors for cardiometabolic diseases (smoking status, physical activity, alcohol consumption, BMI and familial history of type II diabetes mellitus and/or CVD) and motivational aspects, which were divided in practical factors such as time investment, personal beliefs about the own health and lifestyle, and social factors. Physical activity was measured as the number of days per week on which people move at least 30 minutes a day. Alcohol consumption was measured as the number of days per week on which people drink at least six glasses of alcohol a day. For both parts of the prevention program, different motivational aspects were measured. For the health check, we did not measure social aspects, since a health check is completed by the individual himself and therefore no social aspects are involved. All motivational aspects were formulated as statements with response options on a five-point Likert scale. Answering options on the statements were dichotomized because of non-normally distribution. ‘Strongly disagree’, ‘disagree’ and ‘neutral’ were combined into ‘disagree’ and ‘agree’ and ‘strongly agree’ into ‘agree’ for statements regarding the health check. For statements regarding the lifestyle intervention program, the answers ‘not important at all’, ‘not important’ and ‘neutral’ were combined into ‘not important’ and ‘important’ and ‘very important’ were combined into ‘important’.

### Statistical analyses

Univariable and multivariable logistic regression analyses were performed to identify the most important factors related to willingness to participate. Analyses were performed separately for participation in a health check and for participation in a lifestyle intervention program. Variables with a p-value < 0.15 in the univariable analyses were included in the multivariable model. Next, backward logistic regression analyses were applied to remove variables from the multivariable model (p-value > 0.05). Potential collinearity (ρ ≥ 0.5) between the independent variables eligible for the multivariable model was tested before creating the multivariable model. In case of collinearity, the variable with the lowest p-value was added to the model. All statistical analyses were performed using Stata 12.

## Results

**Figure 1:**
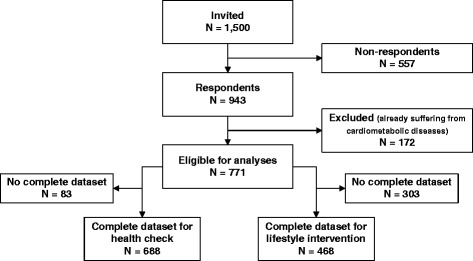
**Number of respondents eligible for the statistical analyses.**

Of the 1,500 invited panel members, 943 returned the questionnaire (response rate 63%). 172 respondents were excluded because they already suffered from a cardiometabolic disease (Figure 1). Socio-demographic and lifestyle characteristics are presented in Table 1.

**Table 1:** **Baseline characteristics**

	**N = 771**
Age (year) (mean ± SD)	53 (16)
Gender	
Female	60%
Education level^a^	
Low	13%
Middle	56%
High	31%
Marital status^a^	
Married / registered partnership	74%
Divorced	6%
Widow(er)	4%
Never married	16%
Smoking status^a^	
Currently smoking	17%
Physical activity: comply with Dutch Standard of Healthy Exercising^a, b^	
No	60%
Alcohol consumption: ≥ 6 glasses per day^a^	
>1 time per week	5%
BMI (kg/m^2^)^a^ (mean ± SD)	25.8 (4.6)
Overweight: BMI 25.0-30.0 kg/m^2^	37%
Obese: BMI ≥ 30.0 kg/m^2^	12%
Familial history of cardiovascular diseases^a^	24%
Familial history of type II diabetes mellitus^a^	19%

^a^No complete number due to missing values.

^b^Dutch Standard of Healthy Exercising: ≥ 30 minutes of moderate intensive exercising on ≥ 5 days per week.

### Participation in health check

Of the respondents, 56% had a positive intention to participate in a health check. In the final multivariable model, seven factors were associated with willingness to participate in health checks (Table 2). Participants aged 65 years and older were more willing to participate compared to the age category 19–38 years (OR = 1.9). Agreeing with the statements ‘Important to know whether I have a high risk’, ‘I want to become aware of healthy lifestyle’ and ‘Due to the check, I have higher chance on aging healthy’ was associated with a positive intention to participate (OR = 4.6, OR = 2.9 and OR = 2.1, respectively). Agreeing with the statements ‘No time to participate’, ‘It makes me unnecessarily worried’ and ‘Feeling healthy, I don’t expect any diseases’ was associated with a decreased willingness to participate (OR = 0.3, OR = 0.3 and OR = 0.7, respectively). Based on the multivariable model, agreeing with all motivating factors and disagreeing with all restraining factors will result in a positive intention to participate of 80 to 90% in all age categories.

**Table 2:** **Predictors of a positive intention to participate in a health check**

		**Univariable**			**Multivariable**		
		**OR**	**95% CI**	**P**	**OR**	**95% CI**	**P**
Age	19-38	1.0			1.0		
	39-55	0.9	0.6-1.4	.68	0.9	0.5-1.5	.61
	56-65	1.3	0.9-2.0	.19	1.4	0.8-2.3	.24
	>65	1.6	1.1-2.5	.03	1.9	1.1-3.3	.02
Gender	Female	1.0	0.8-1.4	.95			
Education level	Low	1.0					
	Middle	1.1	0.6-1.8	.78			
	High	0.9	0.5-1.5	.57			
Marital status	Widow(er)	1.0					
	Divorced	1.4	0.5-3.7	.49			
	Never married	1.6	0.8-3.4	.23			
	Married / partnership	1.1	0.5-2.4	.90			
Smoking status	Currently smoking	0.7	0.5-1.0	.03			
Physical activity: ≥ 30 min/day	≥5 days a week	1.0					
	<5 days a week	1.1	0.9-1.5	.38			
Alcohol cons.^a^: ≥ 6 glasses/day	≤1 time a week	1.0					
	>1 time a week	1.0	0.5-1.9	.97			
Body Mass Index (kg/m^2^)	<25.0	1.0					
	25.0 - 30.0	1.1	0.8-1.5	.74			
	≥30.0	1.0	0.6-1.5	.85			
Fam. hist. of type II DM / CVD^b^	Yes	1.2	0.9-1.6	.23			
*Agree with following statements* ^c^							
Practical factors							
No time to participate		0.2	0.1-0.6	.001	0.3	0.1-1.0	.04
Participation if the insurance pays the check		0.7	0.5-0.9	.01			
Personal beliefs							
It makes me unnecessarily worried		0.2	0.1-0.3	<.001	0.3	0.1-0.4	<.001
Don’t want to give (the risk of) diseases any thoughts^d^		0.3	0.2-0.4	<.001			
Don’t want to take medication due to the check		0.4	0.3-0.5	<.001			
Don’t want to face social consequences of bad outcome		0.6	0.4-0.9	.003			
Don’t want to adjust lifestyle due to the check		0.6	0.3-1.1	.09			
Feeling healthy, I don’t expect any diseases		0.8	0.6-1.0	.09	0.7	0.5-1.0	.04
Important to know how to reduce risk^d^		8.7	5.0-15.2	<.001			
Important to know whether I have a high risk		7.4	5.1-10.6	<.001	4.6	3.0-7.2	<.001
Better to prevent than to cure		6.7	3.2-13.9	<.001			
I want to become aware of healthy lifestyle		6.0	4.3-8.4	<.001	2.9	2.0-4.4	<.001
Important to know whether I have a disease^d^		5.4	3.9-7.5	<.001			
Due to the check I have higher chance on aging healthy		4.1	3.0-5.5	<.001	2.1	1.4-3.0	<.001

^a^Alcohol consumption.

^b^Familial history of type II diabetes mellitus and/or cardiovascular diseases.

^c^Reference category: disagree.

^d^Not included into the multivariable model because of collinearity (ρ ≥ 0.5).

### Participation in lifestyle intervention program

In total, 47% had a positive intention to participate in a lifestyle intervention program. In the final multivariable model, five factors were associated with willingness to participate in a lifestyle program (Table 3). Up to the age of 65 years, a higher age was associated with a higher willingness to participate. A total program duration of six to twelve months increased willingness to participate the most (OR = 9.0). Considering the statements ‘Programs goal is oriented towards preventing relapse’, ‘My general practitioner is informed about my results’ and ‘Program is given to a group’ as important, was associated with an increased willingness to participate (OR = 3.3, OR = 2.6 and OR = 2.0, respectively). Based on the multivariable model, considering all motivating factors as important and using the most optimal program duration, will result in a positive intention to participate of 55 to 80% with the highest percentage in the age category 56 to 65 years.

**Table 3:** **Predictors of a positive intention to participate in a lifestyle intervention program**

		**Univariable**			**Multivariable**		
		**OR**	**95% CI**	**P**	**OR**	**95% CI**	**P**
Age	19-38	1.0			1.0		
	39-55	1.3	0.8-1.9	.25	2.2	1.1-4.1	.02
	56-65	2.2	1.4-3.3	<.001	3.2	1.8-5.9	<.001
	>65	1.2	0.8-1.8	.42	1.6	0.9-2.9	.10
Gender	Female	1.2	0.9-1.7	.17			
Education level	Low	1.0					
	Middle	1.6	0.9-2.7	.11			
	High	1.6	0.9-2.9	.12			
Marital status	Widow(er)	1.0					
	Divorced	2.4	1.0-6.0	.06			
	Never married	1.2	0.6-2.5	.65			
	Married / partnership	1.2	0.8-1.8	.37			
Smoking	Currently smoking	0.6	0.4-0.9	.01			
Physical activity: ≥ 30 min/day	≥5 days a week	1.0					
	<5 days a week	0.9	0.7-1.2	.41			
Alcohol cons.^a^: ≥ 6 glasses/day	≤1 time a week	1.0					
	>1 time a week	0.6	0.3-1.2	.14			
Body Mass Index (kg/m^2^)	<25.0	1.0					
	25.0 - 30.0	0.9	0.6-1.2	.33			
	≥30.0	0.9	0.6-1.4	.62			
Fam. hist. of type II DM / CVD^b^	Yes	1.1	0.8-1.5	.48			
*Importance of topics for decision to participate lifestyle intervention program* ^c^							
Practical factors							
Maximal program duration	No participation anyway	1.0			1.0		
	3 months	3.4	2.0-5.8	<.001	2.5	1.2-5.0	.01
	6 - 12 months	10.7	6.5-17.6	<.001	9.0	4.6-17.5	<.001
	As long as necessary	4.8	2.8-8.2	<.001	3.2	1.6-6.3	.001
Health insurance pays the program		0.8	0.6-1.1	.18			
Course is conducted by internet		0.9	0.7-1.2	.48			
Information and instructions are provided on paper		2.3	1.7-3.2	<.001			
Program is given in the neighborhood		1.8	1.2-2.6	.01			
I can perform the program wherever I want		1.2	0.9-1.6	.27			
Personal beliefs							
Programs goal is oriented towards preventing relapse		4.6	2.8-7.4	<.001	3.3	1.5-6.9	.002
Participation helps me to avoid taking medication		3.0	2.0-4.4	<.001			
Program is focused on setting goals		3.0	2.1-4.3	<.001			
Program is focused on having fun		1.4	1.0-1.9	.03			
Social factors							
My general practitioner is informed about my results		3.0	2.1-4.2	<.001	2.6	1.6-4.4	<.001
Program is given to a group		2.3	1.6-3.4	<.001	2.0	1.1-3.5	.02
My partner is allowed to participate as well		2.0	1.5-2.7	<.001			
Program is given to me individually by the care provider		1.2	0.9-1.6	.26			

^a^Alcohol consumption.

^b^Familial history of type II diabetes mellitus and/or cardiovascular diseases.

^c^Reference category: not important.

## Discussion

Willingness to participate in prevention programs, consisting of a health check and a lifestyle intervention program, was explored in this study. In total, 56% of the participants were willing to participate in a health check. Factors most related to willingness to participate in health checks were age, personal beliefs and having no time to participate. 47% of the participants were willing to participate in a lifestyle intervention program if they turned out to be a person with a high risk for cardiometabolic diseases. Factors most related to willingness to participate were age, program duration and social factors.

This study was aimed at identifying the most relevant factors in participation in prevention programs in people without cardiometabolic disease. Previous studies about participation included patients (at risk for) cardiometabolic diseases and focused on a limited number of factors related to willingness to participate. For the health check, fear of the outcome of the test restrained from participation [21]. For the lifestyle intervention program, mainly social factors motivated to participate [8,28], while time investment was a barrier [15,23]. These motivational aspects were also found as predictors of willingness to participate in prevention programs in our study. Besides the time investment, more practical factors were identified as barriers for participation in lifestyle programs in studies among individuals already suffering from cardiometabolic diseases [15,25]. In our study, these factors were not found as predictors of willingness to participate, which could be explained by the fact that willingness to participate is not equivalent to real participation. Previous studies also found an association between socio-economic status and willingness to participate in both the health check and lifestyle intervention program [15,17]. However, we did not found this association. Motivational aspects outweighed all socio-demographic variables, except age, in our backward regression model.

### Strengths and limitations

Studies about identifying factors that influence willingness to participate in both steps of a population-based prevention program are scarce. To our knowledge, this is the first study that tested the influence of socio-demographic factors, risk factors and three different types of motivational aspects on willingness to participate together in one statistical model.

This study was limited by the amount of missing data, especially concerning education level, marital status and perceived general health. Perceived general health was omitted from the analyses, because this was incorporated in the statements. Omitting marital status and education level from the analyses did not influence the results, since the same multivariable model emerged with and without the use of these factors.

Secondly, mean age and sex distribution in our study slightly differed from the Dutch population. In 2013, 51% of the Dutch population was female and the mean age of the adult population was about 49 years old [30]. In our study, 60% of the participants were female and mean age was 53 years. It is unclear to what extent these differences have influenced our results.

A third limitation is that low risk individuals were incorporated in the analysis of willingness to participate in a lifestyle intervention program, which is meant for high risk individuals. However, the subject of our study was willingness to participate if someone would be eligible for participation in such a program, which is hypothetical because respondents were not told they had a low or high risk of developing cardiometabolic diseases. Therefore, motives and barriers for participation in a lifestyle intervention program were identified for all respondents.

Finally, increasing willingness to participate does not necessarily mean that participation rates will increase or that sustainable participation is guaranteed. However, willingness precedes actual participation, so insight in factors related to willingness to participate gives direction to influencing actual participation in a positive way.

### Implications

Of the participants in our study, 56% and 47% were willing to participate in a health check and a lifestyle intervention program, respectively. By removing barriers to participate, willingness to participate could be increased to 80 to 90%, particularly in the age category 39–65 year, which is the target population for many prevention programs for cardiometabolic diseases.

Barriers for participation in a health check were mainly part of personal beliefs. Providing tailored persuasive information might change personal beliefs, such as feelings of worry and anxiety for knowing the actual risk for cardiometabolic diseases. This could increase willingness to participate. However, providing tailored information is a time-consuming and intensive process. Costs versus benefits of tailoring should be weighted out.

Willingness to participate in a lifestyle intervention program mainly depended on social factors. Informing the general practitioner about results, so that he can discuss these with the individual, and conducting the course in a group are relatively easy to implement.

## Conclusions

About half of the adults without cardiometabolic diseases was willing to participate in either a health check or a lifestyle intervention program to prevent or postpone cardiometabolic diseases, so there is ample room for improvement. Personal beliefs were most strongly related to willingness to participate in a health check, whereas social factors contributed mostly to willingness to participate in a lifestyle intervention program. These findings may form the starting point for increasing participation rates of preventive programs.

## Abbreviations

BMI: Body mass index

## References

[1] World Health Organization (2011). Global Status Report on Noncommunicable Diseases 2010.

[2] Mathers CD, Loncar D (2006). Projections of global mortality and burden of disease from 2002 to 2030. PLoS Med.

[3] Tourlouki E, Matalas AL, Panagiotakos DB (2009). Dietary habits and cardiovascular disease risk in middle-aged and elderly populations: a review of evidence. Clin Interv Aging.

[4] Sattar N, Gaw A, Scherbakova O, Ford I, O’Reilly DS, Haffner SM (2003). Metabolic syndrome with and without C-reactive protein as a predictor of coronary heart disease and diabetes in the West of Scotland coronary prevention study. Circulation.

[5] Repas TB (2007). Challenges and strategies in managing cardiometabolic risk. J Am Osteopath Assoc.

[6] Rice BH, Cifelli CJ, Pikosky MA, Miller GD (2011). Dairy components and risk factors for cardiometabolic syndrome: recent evidence and opportunities for future research. Adv Nutr.

[7] Kraushaar LE, Kramer A (2009). Are we losing the battle against cardiometabolic disease? The case for a paradigm shift in primary prevention. BMC Public Health.

[8] Laws RA, Fanaian M, Jayasinghe UW, McKenzie S, Passey M, Davies GP (2013). Factors influencing participation in a vascular disease prevention lifestyle program among participants in a cluster randomized trial. BMC Health Serv Res.

[9] Ma J, King A, Wilson S, Xiao L, Stafford R (2009). Evaluation of lifestyle interventions to treat elevated cardiometabolic risk in primary care (E-LITE): a randomized controlled trial. BMC Fam Pract.

[10] Unal B, Critchley JA, Capewell S (2005). Modelling the decline in coronary heart disease deaths in England and Wales, 1981–2000: comparing contributions from primary prevention and secondary prevention. BMJ.

[11] National Institute for Clinical Excellence (2010). Prevention of Cardiovascular Disease at Population Level.

[12] Deaton C, Froelicher ES, Wu LH, Ho C, Shishani K, Jaarsma T (2011). The global burden of cardiovascular disease. Eur J Cardiovasc Nur.

[13] Assendelft WJ, Nielen MM, Hettinga DM, van der Meer V, van Vliet M, Drenthen AJ (2012). Bridging the gap between public health and primary care in prevention of cardiometabolic diseases; background of and experiences with the prevention consultation in The Netherlands. Fam Pract.

[14] van der Meer V, Nielen MM, Drenthen AJ, van Vliet M, Assendelft WJ, Schellevis FG (2013). Cardiometabolic prevention consultation in the Netherlands: screening uptake and detection of cardiometabolic risk factors and diseases–a pilot study. BMC Fam Pract.

[15] Lakerveld J, IJzelenberg W, Van Tulder MW, Hellemans IM, Rauwerda JA, Van Rossum AC (2008). Motives for (not) participating in a lifestyle intervention trial. BMC Med Res Methodol.

[16] Elzen H, Slaets JP, Snijders TA, Steverink N (2008). Do older patients who refuse to participate in a self-management intervention in the Netherlands differ from older patients who agree to participate. Aging Clin Exp Res.

[17] Koopmans B, Nielen MM, Schellevis FG, Korevaar JC (2012). Non-participation in population-based disease prevention programs in general practice. BMC Public Health.

[18] Robroek SJ, Van Lenthe FJ, Van Empelen P, Burdorf A (2009). Determinants of participation in worksite health promotion programmes: a systematic review. Int J Behav Nutr Phys Act.

[19] Lambert AM, Burden AC, Chambers J, Marshall T (2012). Cardiovascular screening for men at high risk in heart of Birmingham teaching primary care trust: the deadly trio programme. J Public Health.

[20] Wall M, Teeland L (2004). Non-participants in a preventive health examination for cardiovascular disease: characteristics, reasons for non-participation, and willingness to participate in the future. Scand J Prim Health Care.

[21] Nielsen KDB, Dyhr L, Lauritzen T, Malterud K (2004). You can’t prevent everything anyway: a qualitative study of beliefs and attitudes about refusing health screening in general practice. Fam Pract.

[22] Groeneveld IF, Proper KI, Van Der Beek AJ, Hildebrandt VH, Van Mechelen W (2009). Factors associated with non-participation and drop-out in a lifestyle intervention for workers with an elevated risk of cardiovascular disease. Int J Behav Nutr Phys Act.

[23] Vermunt PWA, Milder IEJ, Wielaard F, Van Oers JAM, Westert GP (2010). An active strategy to identify individuals eligible for type 2 diabetes prevention by lifestyle intervention in Dutch primary care: the APHRODITE study. Fam Pract.

[24] Toft UN, Kristoffersen LH, Aadahl M, von Huth Smith L, Pisinger C, Jørgensen T (2007). Diet and exercise intervention in a general population: mediators of participation and adherence: the Inter99 study. Eur J Public Health.

[25] van Gils PF, Lambooij MS, Flanderijn MH, van den Berg M, de Wit GA, Schuit AJ (2011). Willingness to participate in a lifestyle intervention program of patients with type 2 diabetes mellitus: a conjoint analysis. Patient Prefer Adherence.

[26] Van der Weijden T, Van Steenkiste B, Stoffers HEJH, Timmermans DRM, Grol RPTM (2007). Primary prevention of cardiovascular diseases in general practice: mismatch between cardiovascular risk and patients’ risk perceptions. Med Decis Making.

[27] Schmidt M, Absalah S, Nierkens V, Stronks K (2008). Which factors engage women in deprived neighbourhoods to participate in exercise referral schemes?. BMC Public Health.

[28] Murray J, Honey S, Hill K, Craigs C, House A (2012). Individual influences on lifestyle change to reduce vascular risk: a qualitative literature review. Br J Gen Pract.

[29] Brabers AEM, Reitsma-van Rooijen M, de Jong JD (2012). Consumentenpanel Gezondheidszorg. Basisrapport met informatie over het panel (2012).

[30] Centraal Bureau voor de Statistiek. Bevolking; geslacht, leeftijd en burgerlijke staat, 1 januari. Den Haag/Heerlen. 2014 (www.cbs.nl). 12294181

